# SARS-CoV-2 Vaccination and the Bridge between First and Fourth Dose: Where Are We?

**DOI:** 10.3390/vaccines10030444

**Published:** 2022-03-14

**Authors:** Cristina Stasi, Barbara Meoni, Fabio Voller, Caterina Silvestri

**Affiliations:** 1 Epidemiology Unit, Tuscany Regional Health Agency, Via Pietro Dazzi, 1, 50141 Florence, Italy; barbara.meoni@ars.toscana.it (B.M.); fabio.voller@ars.toscana.it (F.V.); caterina.silvestri@ars.toscana.it (C.S.); 2 MASVE Interdepartmental Hepatology Center, Department of Experimental and Clinical Medicine, University of Florence, 50134 Florence, Italy; 3 Center for Research and Innovation CRIA-MASVE, Careggi University Hospital, 50134 Florence, Italy

**Keywords:** SARS-CoV-2, immunogenicity, vaccine efficacy, COVID-19, variants

## Abstract

The severe acute respiratory syndrome coronavirus 2 (SARS-CoV-2) pandemic has induced the explosion of vaccine research. Currently, according to the data of the World Health Organization, there are several vaccines in clinical (145) and preclinical (195) stages, while at least 10 are already in clinical phase 4 (post-marketing). Vaccines have proven to be safe, effective, and able to reduce the spread of SARS-CoV-2 infection and its variants, as well as the clinical consequences of the development of coronavirus disease-19 (COVID-19). In the two-dose primary vaccination, different time intervals between the two doses have been used. Recently, special attention has been paid to assessing the immunogenicity following booster administration. The third dose of the vaccine against COVID-19 may be administered at least 8 weeks after the second dose. In Israel, a fourth dose has already been approved in immunocompromised groups. The main objective of this review is to describe the principal results of studies on the effectiveness of first-to-fourth dose vaccination to reduce reinfection by variants and the incidence of severe disease/death caused by COVID-19. Vaccines have shown a high level of protection from symptomatic infection and reinfection by variants after a third dose. Accelerating mass third-dose vaccination could potentially induce immunogenicity against variants.

## 1. The State of the Art

In addition to traditional inactivated or live-attenuated vaccines, the context of COVID-19’s emergence has resulted in the need to find a safe vector for antigen and gene delivery and to design nucleic acid-based vaccines (vaccines mRNA and DNA) and subunit vaccines. Many of these new vaccines have been designed by encapsulating nucleic acids or peptides/proteins within polymer and lipid nanoparticles [1]. Regarding vaccines based on inactivated viruses, for safety reasons, the viruses used for vaccines need to be completely inactivated, while viral epitopes inducing protective immunity need to be preserved to allow for the production of high-quality antigens. The virus inactivation process requires a complex procedure, and, although it represents the most cost-effective interventions in medical history, validation of each batch is required to confirm complete inactivation of the recruited pathogens [1,2].

According to the World Health Organization data, there are currently 145 vaccines in clinical development and 195 in preclinical development, while there are RNA-based (*N* = 3), viral vector (*N* = 3), inactivated virus (*N* = 3), and protein subunit (*N* = 1) [3] vaccines in clinical phase 4 (post-marketing).

A recent meta-analysis [4] assessed the vaccine efficacy (from randomized clinical trials and from observed studies) of all vaccines that received or applied for WHO Emergency Use Listing as of 15 August 2021, with at least published data from completed phase 3 studies for the full vaccination course (one or two doses, depending on the vaccine brand). In this meta-analysis, results were only included if appropriate detection occurred 1 week after the final dose. These results demonstrated the high efficacy of COVID-19 vaccines in both clinical trials and real-world settings. The protection against serious disease or death in the general population was at least 80% and often 100%.

At the start of 2021, many countries faced numerous challenges during the early stages of mass vaccination. In March 2021, the European Center for Disease Prevention and Control (ECDC) published a report [5] providing a snapshot of the current situation on the basis of responses to vaccine questions posed to the Member States. In total, the vaccine questions were answered by 28 EU/EEA countries (except Bulgaria and Slovenia). Almost all countries responded that the timing of vaccine deliveries is unpredictable and is often changed by vaccine manufacturers, thus having a significant impact on planning efficiency. Almost half of the countries (*N* = 12) have adopted effective strategies to limit the waste of unused vaccines. In particular, seven countries are vaccinating people outside the target groups to avoid wasting doses. In Belgium, reserve lists were created that drew on other groups to be vaccinated to address the problem of some health workers’ reluctance to vaccination. Lists of reserve persons have also been developed in Italy and Croatia, while, in Austria, a list with subjects with priority for vaccination has been created for last-minute vaccinations. As mass vaccination required an increase in skilled staff to administer the vaccines, six countries (22%) reported staff shortages/lack of skilled labor, and four countries (15%) reported the need to train additional (unqualified) personnel. Against this background, the recruitment and training of more medical and nonmedical personnel were considered essential for the progression of vaccination campaigns. Ten countries (36%) highlighted a lack of equipment needed for vaccination, while 21% of countries reported disinformation, particularly regarding the vaccination priority of certain groups and the prioritization method. Vaccination hesitancy issues vary from country to country and are related to specific local social and cultural contexts.

In February and March 2021, most vaccines used a two-dose protocol with some variation in the time interval between the two doses. The BNT162b2 mRNA vaccine was authorized for the administration of two doses at an interval of 3 weeks, while the interval for the adenovirus-based ChAdOx1 (AZD1222 (AstraZeneca BioPharmaceuticals, Cambridge, UK)) vaccine was longer, as many studies showed a longer interval to increase its effectiveness. Although both the mRNA and the ChAdOx1 vaccines show high clinical efficacy, great attention has been paid to assessing their immunogenicity after a single administration. This is of particular interest concerning their use in elderly people, where immune senescence can potentially act by limiting their immune response. The BNT162b2 mRNA (Pfizer BioNTech, New York, NY, USA) and adenovirus-based ChAdOx1 (AstraZeneca) vaccines present antigens in different modalities, and this may be reflected in a different profile or extent of humoral immunity or adaptive response.

In July 2021, the first country in the world to administer three doses of BNT162b2 in the general population aged ≥60 years was Israel. A third dose was administered 5 months after the two doses, inducing a 5–7-fold increase in neutralization titers and an 11.3-fold reduction in infections [6]. In the UK, the booster was initially limited to immunocompromised subjects before the extension to the broader population aged 50 years [7].

In January 2022, international regulatory authorities [8] published a report summarizing the main global response strategies to the Omicron variant of SARS-CoV-2. By reviewing data on the impact of Omicron, international regulatory authorities concluded that current vaccines offer less protection against infections and mild illnesses caused by this variant; however, after a booster dose, vaccination continues to offer significant protection against the severe forms of COVID-19. Real-world evidence from studies conducted in Canada and the United Kingdom suggests that, despite the decline in vaccine efficacy against Omicron after the primary series, a booster dose (homologous or heterologous) is capable of restoring high levels of protection in the period immediately following vaccine administration. Protection from hospitalization is very high after a booster dose (up to 90% according to UK data), although it is still necessary to determine, with longer follow-up, if this level of protection is maintained.

In Israel [9], national data from the second week of December 2021 revealed a reduced vaccine efficacy against infections and hospitalizations in all age groups, attributable to a combination of declining immunity and cases due to the spread of Omicron. Israel has recently started administering a four-dose of vaccine to people ≥65 years of age and with comorbidities to overcome the new outbreak due to Omicron.

Currently, vaccine booster uptake in the total population in the EU/EEA is about 50% [10].

Despite the difficulties and differences encountered in the various countries of the world, we describe here the main results of research studies on the effectiveness of the first, second, and third doses of the vaccine, with the hope that more and more people can be vaccinated and, consequently, vaccination can put an end to this nightmare.

## 2. Safety and Efficacy of Single Dose in Subjects with or without Previous SARS-CoV-2 Infection

Saadat et al. [11] investigated whether a single dose of an mRNA-based COVID-19 vaccine could elicit immune responses in healthcare workers with previous COVID-19 infection. This study enrolled healthcare workers who previously underwent a hospital-wide serological assessment at the University of Maryland Medical Center. Out of 59 healthcare workers enrolled voluntarily, 17 were anti-SARS-CoV-2 IgG-negative (mean age 38 years, 71% women), 16 were COVID-19 asymptomatic IgG-positive (average age 40 years, 75% women), and 26 were IgG-positive with a history of symptomatic COVID-19 (mean age 38 years, 88% women). Subjects were stratified into three groups: anti-SARS-CoV-2 IgG-negative and IgG-positive with or without a history of symptomatic COVID-19. BNT162b2 (Pfizer/BioNTech) or mRNA-1273 (Moderna) vaccines were administered on the basis of vaccine preference or availability. At 0, 7, and 14 days, the median antibody titers were higher in the asymptomatic (COVID-19 asymptomatic IgG-positive) and symptomatic group (IgG-positive with history of symptomatic COVID-19) compared to the anti-SARS-CoV-2 IgG-negative group. Krammer et al. [12] described the antibody responses in 109 individuals with and without prior SARS-CoV-2 infection (68 without prior SARS-CoV-2 infection or seronegative; 41 with prior SARS-CoV-2 infection or seropositive) who received the first dose of vaccine (BNT162b2/Pfizer; mRNA-1273/Moderna) in 2020. Seronegative individuals, within 9–12 days of the first dose, showed low immune responses of vaccination. In contrast, within 5–8 days of vaccination, individuals with pre-existing SARS-CoV-2 showed high antibody titers. The antibody titers of vaccinates with pre-existing (seropositive) immunity were 10–20 times higher than those in subjects without pre-existing immunity after the first dose, and they were 10 times higher in seropositive subjects compared with seronegative subjects after the second dose of vaccine. This study also compared the frequency of local and systemic reactions after the first vaccine dose in 231 individuals (148 seronegative and 83 seropositive). The most common symptoms were pain, swelling, and erythema at the injection site, which took place regardless of the serological status at the time of vaccination and resolved spontaneously a few days after vaccination. Systemic side-effects such as fatigue, headache, chills, fever, and muscle or joint pain were higher in seropositive than seronegative subjects. These results suggest that a single dose of mRNA vaccine induces very rapid immune responses in seropositive individuals. The post-vaccine antibody titers were comparable to or higher than the titers found in seronegative individuals who received two vaccine doses. Furthermore, in phase 3 vaccine studies, the reactogenicity after both the first and the second dose was substantially more marked in seropositive subjects, suggesting that a single dose may be sufficient in subjects with prior infection.

The study by Parry et al. [13] evaluated immune responses in an elderly population (≥80 years) who received a single dose of BNT162b2 mRNA vaccine (Pfizer) or adenovirus-based ChAdOx1 nCoV-19 vaccine (AZD1222) (AstraZeneca). Out of 165 subjects enrolled, 76 received the BNT162b2 (Pfizer) vaccine (mean age 84 years) and 89 received the ChAdOx1 (AstraZeneca) vaccine (median age 84). Antibody responses were observed in 93% of subjects after administration of the BNT162b2 (Pfizer) vaccine and 87% after administration of the ChAdOx1 (AstraZeneca) vaccine. Corresponding antibody titers measurements after 5–6 weeks were 19.3 and 19.6 U/mL (median), respectively. Responses were also assessed using the spike-specific quantitative ELISA test (Abbott) with responses not showing statistically significant differences between BNT162b2 and ChAdOx1. Of eight subjects who showed a previous natural infection based on N-specific positivity to serology, five subjects received BNT162b2 and three received ChAdOx1. Regarding the induction of specific T-cell responses, cell responses were detectable in 12.3% after 5 weeks from a single dose of the BNT162b2 vaccine and in 30.7% after 5 weeks from a single dose of ChAdOx1 vaccine. In addition, the extent of cellular responses was three times greater in the ChAdOx1 (AstraZeneca) subgroup than that induced by BNT162b2 (Pfizer), a difference that persisted even after the exclusion of subjects with natural infection. These results demonstrate that reassuring levels of humoral immunity against primary infection exist after the administration of both types of vaccines. However, the adenovirus-based vaccine (ChAdOx1 AstraZeneca) induced a stronger cellular immune response than vaccination with BNT162b2 mRNA vaccine (Pfizer).

In general, the side-effects of the two mRNA vaccines are more pronounced after the second dose, whereas, for AstraZeneca, stronger side-effects are observed after the first dose.

Sadoff et al. [14] evaluated the safety and efficacy of a single dose of Ad26.COV2.S (5 × 10^10^ viral particles) for COVID-19 prevention as part of an ongoing phase 3 study (ENSEMBLE) and SARS-CoV-2 infection in adult subjects, reporting the results of the preliminary analyses. This 2 year, multicenter, randomized, double-blind, placebo-controlled, phase 3 pilot study was conducted in Argentina, Brazil, Chile, Colombia, Mexico, Peru, South Africa, and the United States. Randomization (1:1) was performed with a web response system that assigned Ad26.COV2.S (Janssen Vaccines & Prevention BV, Leiden, The Netherlands) or saline placebo stratifying by research center, age groups, and the presence/absence of coexisting conditions associated with an increased risk of severe COVID-19. The vaccine (5 × 10^10^ viral particles) or placebo was administered via the intramuscular route (0.5 mL) on day 1 by a healthcare professional (blinded). Participants electronically reported any COVID-19 symptoms using the COVID-19 Symptoms Questionnaire. The primary endpoints were the evaluation of the efficacy and safety of the vaccine against COVID-19 with an onset at least 14 and 28 days after vaccine administration in the population that tested negative for SARS-CoV-2 infection. A total of 39,321 subjects were SARS-CoV-2-negative, of whom 19,630 were assigned to receive Ad26.COV2.S and 19,691 were assigned to receive a placebo. Subjects were followed for a median of 58 days (range: 1–124 days), and 55% of subjects were followed for at least 8 weeks. The results of the study showed that the subjects who received the Ad26.COV2.S vaccine were protected with an efficacy of 66.9% against moderate to severe–critical COVID-19 (116 cases in the vaccine group vs. 348 in the placebo group) at least 14 days after vaccine administration; an efficacy of 66.1% (66 cases in the vaccine group vs. 193 in the placebo group) was registered at least 28 days after administration. After at least 14 days and 28 days, efficacies of 76.7% and 85.4%, respectively, were recorded against severe-critical COVID-19. Although the 20H/501Y.V2 variant represented 94.5% of cases in South Africa, the vaccine efficacy was 52.0% and 64.0% against moderate to severe COVID-19 at least 14 days and 28 days after administration, respectively; the efficacy against severe-critical COVID-19 was 73.1% and 81.7%, respectively. In the Ad26.COV2.S vaccine group, adverse events (generally mild to moderate) were transient and occurred more frequently compared to the placebo group. In the group receiving the vaccine, three deaths were reported, none related to COVID-19, while there were 16 deaths in the placebo group, five related to COVID-19. The results of the study showed that a single dose of Ad26.COV2.S was sufficient to provide protection against symptomatic COVID-19, particularly against serious critical illnesses (hospitalization and death), even in countries where the variants were prevalent.

During 12 months of follow-up, Hall et al. [15] studied the association between the presence of SARS-CoV-2 antibodies and a reduction in the risk of symptomatic and asymptomatic reinfection. The SIREN study (SARS-CoV-2 Immunity and Reinfection Evaluation) was a prospective cohort study conducted across the UK among staff working in publicly funded National Health Service (NHS) hospitals. The study enrolled all healthcare professionals, as well as support and administrative staff, working in the participating hospitals, capable of providing written informed consent and being able to be followed up for 12 months. At baseline and every 2 weeks, questionnaires were sent electronically, collecting information on symptoms and exposure to SARS-CoV-2. SARS-CoV-2 antibody testing (every 4 weeks), and real-time PCR nucleic acid amplification testing (NAAT) (RT-PCR) (every 2 weeks) was performed at enrollment and at regular intervals. Reinfections were classified as confirmed, probable, or possible on the basis of serological, genetic, and virological tests. Subjects with two positive PCR samples 90 days or more apart or seropositive subjects with a new positive PCR test at least 4 weeks after the first antibody positivity were defined as possible cases of reinfection. From 18 June 2020 to 31 December 2020, out of 30,625 subjects, 51 withdrew, 4913 were excluded, and 25,661 participants were enrolled. In the positive cohort (positivity at enrollment or positive antibodies from previous clinical laboratory samples, with or without a previous positive PCR test; negative antibodies at enrollment with a positive PCR result before enrollment), consisting of 8278 participants, 155 infections were detected. In the negative cohort (negative antibody test and no previous and documented positive PCR or antibody test) consisting of 17,383 participants, 1704 new infections were found with a positive PCR. Regarding primary infections, 1369 (80.3%) of these cases were symptomatic of the infection, 1126 (66.1%) had typical symptoms of COVID-19, 243 (14.3%) had other symptoms, 293 (17.2%) were asymptomatic, and 42 (2.5%) did not complete the questionnaire by the time symptoms were present. Regarding reinfections, 78 (50.3%) were symptomatic and 50 (32.3%) had typical symptoms of COVID-19. Data analysis showed that a previous SARS-CoV-2 infection had a protective effect (84% lower risk of infection) for the subsequent 7 months.

These findings suggest that a single-dose vaccination strategy could be adopted for individuals with prior COVID-19 or they could be placed further down the vaccination priority list on the basis of the antibody titer.

Table 1 shows the main studies on vaccine efficacy.

## 3. Immune Response after the Second Dose

In all countries, health workers, being at the greatest risk of contracting the infection, were vaccinated first. Fabiani et al. [16] evaluated, through the COVID-19 surveillance database, the real-life efficacy of the BNT162b2 mRNA (Comirnaty) vaccine in a cohort of health workers from the province of Treviso and the Veneto region (Italy). Of 6423 healthcare workers (mean age = 47.1 years ± 10.8, F = 56.6%) included in the analysis, there were 56.5% nurses, 22.9% medical doctors, and 20.6% social healthcare workers. Screening for healthcare professionals was conducted every 8 days and additionally at any time for symptoms compatible with COVID-19. The vaccination campaign started on 27 December 2020, and, by 24 March 2021, 147 (2.3%) subjects had received one dose and 5186 (80.7%) had received two doses of the BNT162b2 mRNA vaccine (median time between the two doses was 22 days). The vaccination completion rate (both doses) was higher in physicians (85.7%) and overall in health personnel working in hospitals (82.1%). Compared to unvaccinated healthcare workers, the cumulative probability of developing SARS-CoV-2 infection in healthcare workers was constantly reduced in subjects who had received at least one dose of the BNT162b2 mRNA (Comirnaty) vaccine. The vaccine efficacy for the prevention of symptomatic and asymptomatic SARS-CoV-2 infection from 14–21 days after the first dose was estimated at 84% and at 83% for the prevention of the symptomatic form; 7 days after the administration of the second dose, the vaccine efficacy was instead 95% and 94% for the two groups, respectively. Furthermore, despite an increased risk of exposure for healthcare workers due to increased hospital admissions for COVID-19, new COVID-19 cases remained stable at a long-term immunization rate of approximately 70%.

SARS-CoV-2, like other viruses, mutates over time. Some of these mutations may have little or no impact on the properties of the virus, while some changes may affect the properties of the virus, such as the ease of spread, the severity of the disease, or the performance of vaccines, drugs, diagnostic tools, or other measures, thus affecting public and social health.

The WHO [17] has been monitoring since January 2020 the emergence of variants that pose a greater risk to global public health, urging the characterization of specific variants of interest (VOIs) and variants of concern (VOCs). VOCs are characterized by an increase in transmissibility or a detrimental change in the epidemiology of COVID-19, increased virulence or a change in the clinical presentation of the disease, or a decrease in the effectiveness of public health and social measures or the availability of diagnostic, vaccine, and therapeutic tools. The first VOC (B.1.1.7), designated by WHO as Alpha, was earliest documented in late 2020 in the UK and displayed increased transmissibility [18]. It is, therefore, probable that the selection of this variant occurred to improve the binding capacity of the virus to the ACE2 receptor and its transmissibility. Considering the viral dynamics and viral evolution in response to different selection pressures in an immunocompromised individual, one hypothesis of the emergence of the Alpha variant was that it accumulated many mutations within a chronically infected immunocompromised patient [19].

Emary et al. [20] conducted an in vitro analysis of vaccine-induced neutralizing antibodies against variant B.1.1.7 and a clinical analysis of the ChAdOx1 nCoV-19 (AstraZeneca) efficacy against the disease caused by this variant, which has caused much concern in the UK. This single-blind, multicenter, randomized (1:1 ratio receiving the standard dose ChAdOx1 nCoV-19 vaccine or a meningococcal group A, C, W, and Y conjugate (MenACWY) control vaccine), phase 2/3 study enrolled only subjects ≥18 years with a high risk of exposure to SARS-CoV-2, such as those operating in health and social care settings. The study was conducted in 19 research centers in England, Wales, and Scotland between 31 May and 13 November 2020. The booster doses were given between 3 August and 30 December 2020. A total of 2773 subjects received the first low-dose vaccine (2.2 × 10^10^ viral particles). They provided weekly self-collected nose and throat swabs for a nucleic acid amplification test (NAAT) by kits provided by the Department of Health and Social Care 1 week after the first dose, using home testing. Participants were invited weekly by email or SMS to contact the study team in the event of the onset of COVID-19 symptoms (cough, fever above 37.8 °C, shortness of breath, anosmia, or ageusia). Neutralizing antibody responses were measured using a live virus microneutralization test against the B.1.1.7 line and a non-B.1.1.7 canonical variant (BetaCoV/Australia/VIC01/2020). Of 8534 participants in the primary efficacy cohort, 59% were women and 78% were aged between 18 and 55. A total of 520 cases of SARS-CoV-2 infection were observed between 1 October 2020 and 14 January 2021, from NAAT-positive nasal and pharyngeal swabs collected from 1466 subjects. A total of 401 swabs, collected from 311 participants, were successfully sequenced. The clinical efficacy of the vaccine against symptomatic, positive NAAT infection was 70.4% for B.1.1.7 and 81.5% for the non-B.1.1.7 variant. For asymptomatic cases or cases of unknown infection, detected through weekly swabs, vaccine efficacy was greater for non-B.1.1.7 infections (69.7%) than for variant B.1.1.7 (28, 9%), although few cases were available for analysis. Overall efficacy against asymptomatic cases or cases of unknown infection was 61.7% (36.7 to 76.9) against variant B.1.1.7 and 77.3% (65.4 to 85) against the other variants. Vaccine efficacy against variant B.1.1.7 was 66.7% in the standard-dose group compared to 77.9% in the low-dose group, while, for cases in the non-B.1.1.7 variant group, efficacy was 78.0% in the standard-dose group compared to 87.2% in the low-dose group. When cases were stratified by standard-dose and low-dose vaccine, few asymptomatic cases or cases of unknown infection were found, which did not allow for robust comparisons. The in vitro experiments showed that the ChAdOx1 nCoV-19 vaccine has reduced neutralization activity against variant B.1.1.7 compared to a non-B.1.1.7 variant.

Vaccination with ChAdOx1 nCoV-19 could also induce a reduction in viral load with a probable reduction in the transmission of the infection.

Suburraro et al. [21] evaluated antibody responses approximately 3 weeks after the first or second dose of BNT162b2 mRNA (Pfizer/BioNTech, Mainz, Germany) in the first 185 adults aged 70 to 90 years, recruited from the end of January 2021 through primary care networks in north London, as part of the national program. The antibody responses of the vaccinated subjects were also compared to those from 100 samples collected approximately 3–6 weeks after symptom onset in subjects affected by mild to moderate COVID-19, PCR-confirmed. Collected samples were tested for two antibodies to nucleoprotein (N), identifying a previous SARS-CoV-2 infection, and for three antibodies to the spike protein (S) evaluating the vaccine response. In the cohort of 185 subjects, 15 were positive for nucleoprotein antibodies, 99 were enrolled after the first dose of BNT162b2 mRNA (Pfizer/BioNTech) vaccine, and 86 subjects were enrolled after receiving two doses 3 weeks apart. In subjects receiving their first dose of vaccine (*n* = 99), sera were collected on days 0 and 18 and 33 days after; in subjects receiving two doses (*n* = 86), sera were collected only between the 21st and 25th days after the second dose. All 86 subjects were positive for antibodies against the spike protein in all three tests. Antibody levels after a single dose of vaccine in previously uninfected individuals were lower than in convalescent sera. After two doses of the vaccine, antibody titers were significantly higher in subjects without previous SARS-CoV-2 infection than those found in convalescent sera. Indeed, there was no evidence of an increased antibody response following the second dose of the vaccine in those previously infected with SARS-CoV-2. On quantitative antibody tests, titers were slightly lower among subjects ≥80 years of age compared to those aged 70 to 79 years, but the difference was not statistically significant. 

In the UK, the interval between COVID-19 vaccine doses from the authorized 3–4 weeks up to 12 weeks was extended by the Joint Committee on Vaccination and Immunization, to maximize the number of subjects with a single dose of vaccine. This was based on the real-world efficacy results of COVID-19 vaccines demonstrating high protection, after a single dose, against SARS-CoV2 infection, COVID-19, hospitalization, and deaths in the UK elderly population [22], and this was confirmed in the population of Israel achieving mass vaccination [23].

Edara et al. [24] evaluated the antibody response against four variants of SARS-CoV-2 and determined whether the mutations within the spike protein are associated with the neutralization of the virus after infection or vaccination. Samples from three groups of subjects were examined: 20 patients with COVID-19 (5–19 days after the onset of symptoms); 20 convalescent subjects (32–94 days after the onset of COVID-19 symptoms); 14 healthy subjects (aged 18–55 years) who received two doses of the mRNA-1273 vaccine (Moderna). Adults (mean age = 57 years, range: 26–85) admitted for COVID-19 were randomly selected. Samples were collected in the first 9 days (range: 2–9) of their hospital stay (range: 3–33 days) and mostly 1–2 weeks after symptom onset (range 5–19 days). Most of these patients (*N* = 16) had comorbidities, 19/20 patients had severe disease, and one patient had a moderate disease. Four variants were examined, including some very similar to the parent SARS-CoV-2 strain (A.1) and emerging variants with mutations in the spike protein (B.1, identified in Georgia; B.1.1.7, identified in the United Kingdom; N501Y SARS-CoV-2, generated from an infectious clone). Despite the small sample size and possible selection bias, this study demonstrated that, against SARS-CoV-2 variants, including B.1, B.1.1.7, and N501Y, antibody neutralizing activity induced by both vaccine and infection is present. In the event of other additional variants, neutralizing antibody responses should be monitored both after infection and after vaccination.

Hung et al. [25] reported the results of trials and clinical studies conducted to evaluate the effectiveness of the ChAdOx1 nCoV-19 vaccine (AZD1222) (Oxford–AstraZeneca) after two standard doses administered at intervals of 12 weeks or more. The authors pointed out that a report based on an interim analysis of four randomized controlled trials (conducted in Brazil, South Africa and the United Kingdom) found an overall efficacy of the vaccine of 70.4%. The efficacy was greater than 90% in subjects receiving a low dose (2 × 10^10^ viral particles per dose) followed by a standard dose (5 × 10^10^ viral particles); on the contrary, vaccine efficacy was 62.1% in subjects receiving two standard doses (4 weeks apart). Out of 17,178 participants (56.4% women), vaccine effectiveness of 76.0% was found from day 22 (59.3–85.9) after a single standard dose, and antibody levels were conserved through day 90, with a minimal decline during this period. The efficacy of the vaccine was significantly higher (81.3%) after two standard doses administered at intervals of 12 weeks or more, compared to 55.1% for administration at less than 6 weeks. The authors highlighted important limitations of these studies, including a lack of prospective design of the study or randomization. Moreover, only one of the four studies was double-blind; baseline characteristics between the cohorts receiving a single dose and the cohorts receiving two doses were substantially different (older mean age, different distribution of gender and ethnicity, and a lower percentage of health or social workers in the cohorts using two doses than in cohorts using the single dose). Despite these limitations, a single dose of the ChAdOx1 nCoV-19 vaccine was found to be highly effective 90 days after the first dose, and a longer interval of administration between the first and second dose induced higher efficacy and vaccine protection against symptomatic COVID-19.

Voysey et al. [26] reported the results of clinical trials conducted to evaluate the efficacy of the ChAdOx1 nCoV-19 vaccine (AZD1222) (Oxford–AstraZeneca) vaccine after two standard doses administered at intervals of 12 weeks or more. In particular, the authors pointed out that an interim analysis of four randomized controlled trials carried out in Brazil, South Africa, and the United Kingdom found an overall efficacy of the vaccine of 70.4%, with a superior efficacy of 90% in those who received a low dose (2 × 10^10^ viral particles per dose) followed by a standard dose (5 × 10^10^ viral particles) and a vaccine efficacy of 62.1% (95% CI 41, 0–75.7) in those who received two standard doses (4 weeks apart). To achieve maximum public health benefit, the UK government decided to give the first doses to as many people as possible, with an interval between the first and second dose of up to 12 weeks. The studies showed that, out of a total of 17,178 participants (56.4% women), the efficacy of the vaccine after a single standard dose was 76.0% (59.3–85.9) starting from day 22. Antibody levels were maintained through day 90, with a minimal decline during this period. Vaccine efficacy was higher (81.3%) after two standard doses administered at intervals of 12 weeks or more, compared with 55.1% of the dose administered at less than 6 weeks. 

A study by Kuodi et al. [27] investigated the association between vaccination status and the long-term reporting of COVID-19 symptoms. Subjects of age ≥18 tested for SARS-CoV-2 infection by RT-PCR between 15 March 2020 and 15 November 2021, in three major hospitals in northern Israel (Ziv Medical Center, Padeh-Poriya Medical Center, and Galilee Medical Center), were invited, between 16 July and 18 November 2021, with a link to a questionnaire available online in the most common languages in Israel (Hebrew, Arabic, Russian, and English). Of the 79,482 subjects invited to participate in the study, 3562 (4.5%) subjects aged ≥18 agreed to participate: 2437 had no previous SARS-CoV-2 infection, while 1125 had a prior infection; 174 infected individuals were excluded because they did not report their vaccination status. Among 951 infected subjects included in the study, 67% had at least one symptom at diagnosis of COVID-19, 36% received one dose, and 31% received at least two doses. Among the unvaccinated, 69% reported at least one symptom at diagnosis of COVID-19, compared with 57% of those who received two doses and 74% of those who received only one dose. Compared to individuals vaccinated with one/two doses, unvaccinated subjects were younger, reflecting COVID-19 vaccination patterns in the general population. The median time between acute illness and symptoms was 8 months in the unvaccinated group and 4 months in subjects receiving two doses. Regarding post-COVID-19 symptom reporting, 337 (35%) of the 951 infected subjects (vaccinated and unvaccinated) reported not fully recovering from initial COVID-19 symptoms at follow-up. The most commonly encountered symptoms were fatigue (22%), headache (20%) weakness in the arms or legs (13%), and persistent muscle pain (10%). Subjects receiving two doses were 36–73% less likely to report eight of the 10 most commonly reported symptoms. Symptoms were mainly present in the older age groups, particularly in those aged >60. This study demonstrates the vaccine efficacy against COVID-19 also against the long-term effects of SARS-CoV-2 infection.

Table 1 shows the main studies on vaccine efficacy.

## 4. From the Third Dose to the Fourth Dose

In November 2021, Juno and Wheatley [28] addressed the issues concerning the need to maintain protection against emerging variants and to increase the efficacy of the vaccine in vulnerable populations including subjects with primary immunodeficiencies, subjects undergoing immunosuppressive therapies, and the elderly. The study by Choi et al. [29] evaluated the immune response after two doses of mRNA-1273 (Moderna) and, 6 months later, after a third (booster) dose of the same vaccine, but reformulated against the Beta variant (B.1.351) or a multivalent vaccine containing both spike sequences. The decrease in neutralization activity of the initial two-dose vaccination was restored by boosters that were found to safely and effectively increase protection against variants. In subjects receiving the booster, the Beta variant, known to escape vaccine-induced antibody responses, was only slightly present. 

A study by Shroff et al. [30] investigated the vaccination of cancer patients, noting that a reduced immune response in these patients could be related to the time elapsed since the last cytotoxic treatment. In these subjects, the administration of a third dose of the vaccine improved the neutralizing antibody responses in the majority of patients (16 out of 20).

In July 2021, Israel became the first country in the world to have three doses of BNT162b2 administered in the general population aged ≥60 years. For people vaccinated 5 months earlier, a third dose induced a 5–7-fold increase in neutralization titers and an 11.3-fold reduction in infections. In the UK, the booster dose was initially limited to immunocompromised individuals before being extended to the broader population over 50 years old. In Israel, Patalon et al. [31] evaluated the short-term efficacy of a two-dose vaccine schedule with an mRNA vaccine BNT162b2 (Pfizer/BioNTech) compared to the three-dose schedule in subjects ≥40 years. This case-control study was conducted using data from Maccabi Healthcare Services (MHS). The analyses covered the period from 1 August 2021 (when the booster dose was widely administered among eligible individuals) to 4 October 2021. Subjects were only eligible for a booster dose at least 150 days after the second dose. This study, therefore, estimated the decrease in the likelihood of finding a positive test result at different time intervals after administration of the third dose (0–6, 7–13, 14–20, 21–27 and 28–65 days) compared with those who had only received two doses. During the study period, 232,500 PCR tests for SARS-CoV-2 were performed among 306,710 subjects 40 years of age or older (55% female) who had no documented prior SARS-CoV-2 infection. In the group that received two doses, out of 227,380 total tests performed, 14,989 were positive (6.6%), while, in the group that received three doses, out of 272,852 total tests performed, 4941 were positive (1.8%). The marginal benefit of the efficacy of the third dose compared to the second dose gradually increased over time, with a moderate marginal benefit found from day 7 to day 13 (58%) and a high marginal benefit from day 14 to day 20 and beyond (85%). The marginal benefit was similar in all age groups after the first 2 weeks; moreover, the benefit was similar regardless of the presence of the comorbidities. Similarly, the case-control analysis estimated that the marginal measure of the efficacy of the booster dose compared to the second dose increased from 50% after 6 days after the booster to 71% between 7 and 13 days after the booster, 87% between day 14 and day 20, 85% between day 21 and day 27, and 83% between day 28 and day 65 after the booster. The odds of hospitalization among subjects receiving a booster dose were 92% to 97% lower than subjects receiving only two doses. Therefore, the BNT162b2 vaccine was associated with a lower likelihood of SARS-CoV-2 infection and hospitalization. Further data are needed to determine the length of immunity after the booster dose.

A clinical trial entitled “A Study to Evaluate the Immunogenicity and Safety of mRNA-1273.211 Vaccine for COVID-19 Variants” (ClinicalTrials.gov Identifier: NCT04927065) [32] was conducted by the biotech company Moderna. This trial, designed to anticipate and contrast some mutations such as those that later emerged in the Omicron variant, involves the evaluation of both the prototype vaccine (mRNA-1273) and the multivalent vaccine candidates (mRNA-1273.211, mRNA-1273.213), incorporating variants of previous variants of concern (VOC). The mRNA-1273.211 vaccine includes several mutations of the Omicron variant already present in the Beta variant. For this type of vaccine, Moderna completed the safety and immunogenicity study for the doses of 50 µg (*N* = 300) and 100 µg (*N* = 584). On 20 December 2021, Moderna published the preliminary results [33] of the 50 µg mRNA-1273 booster dose trial against the Omicron variant, which showed an approximately 37-fold increase in neutralizing antibody levels after 29 days of the booster dose against Omicron compared to the pre-boost levels, while a 100 µg dose of mRNA-1273 increased neutralizing antibody levels approximately 83-fold compared to pre-boost levels. The mRNA-1273.213 vaccine includes many mutations that are present in the Omicron variant, as well as in the Beta and Delta variants; it has already been tested for the 100 µg dose (*N* = 584) and is being studied for the 50 µg dose in 584 other subjects.

Multivalent vaccines have the potential to induce high levels of specific neutralizing antibodies against the Omicron variant at both 50 µg and 100 µg doses. Given the rapid spread of the Omicron variant and the greater complexity of implementing a new vaccine, the company is focusing most short-term efforts on countering the Omicron variant with the booster dose of mRNA-1273; at the same time, in the coming months it will seek to evaluate the levels and duration of neutralizing antibodies induced by multivalent candidates, including a specific vaccine (mRNA-1273.529) for Omicron. Doria-Rose [34] evaluated the potential impact of the Omicron variant on vaccines using serum samples from subjects administered the mRNA-1273 vaccine. In particular, when the neutralizing activity of the antibodies induced by vaccination was tested on serum samples obtained 4 weeks after the administration of two doses of vaccine (100 µg of mRNA-1273), the Omicron variant was 41–84 times less sensitive to neutralizing activity of antibodies compared to the English variant (D614G) and 5.3–7.4 times less sensitive than the Beta variant. A booster dose of 50 µg increased the neutralization activity of antibodies (by about 12 times) against the Omicron variant and was able to significantly reduce the risk of symptomatic infections.

On 2 January 2022, Israel recommended a fourth dose for immunocompromised groups, and the country also offered a fourth dose to healthcare workers and people older than 60 years.

Gruell et al. [35] evaluated the neutralizing activity of antibodies against the Omicron variant in vaccinated and convalescent subjects. The vaccine-induced neutralizing activity was tested against Wu01 (parent virus), Alpha (B.1.1.7), Beta (B.1.351), Delta (B.1.617.2), and Omicron. All the samples showed high titers of neutralizing activity, at an inhibitory dilution of 50%; specifically, a titer of 546 was recorded against the Wu01 strain, while it was reduced to 331 against Alpha, 172 against Delta, and 40 against Beta. In particular, only eight of the 30 vaccinated individuals (27%) showed a detectable neutralizing serum activity against Omicron, but it was significantly lower than the Beta variant (*p* < 0.0001). After two doses of BNT162b2, the neutralizing activity against Wu01 decreased fourfold in the 5 month period (from 546 to 139), but it was strongly increased after the booster dose (to 6241). Neutralizing activity against the Omicron variant after two doses of vaccine remained low but increased more than 100-fold following administration of the booster dose of BNT162b2 (1195), and it was higher than that recorded against Wu01 after the second dose of BNT162b2. Similarly, the neutralizing activity in the convalescent subjects of COVID-19 was determined. Immediately after infection, the neutralizing activity against Wu01 was variable (37 to 11,008). Lastly, Gruell et al. [35] studied the relationship between the high number of mutations in the Omicron variant and the activity of neutralizing SARS-CoV-2 monoclonal antibodies shown to effectively reduce COVID-19 associated morbidity and mortality. Nine monoclonal antibodies were tested against the Wu01 strain and variants Alpha, Beta, Delta, and Omicron, including antibodies authorized for clinical use (bamlanvimab, etesevimab), REGN10933 (casirivimab), REGN10987 (imdevimab), and S309 (sotrovimab), an antibody in clinical study phase (DZIF-10c), and additional antibodies representative of different classes of antibodies (P2B-2F6, C102, and Fab2-36) that target the SARS-CoV-2 receptor-binding domain (RDB), i.e., the protein necessary for the first binding of SARS-CoV-2 protein S to the human ACE2 cell receptor to occur. All antibodies showed neutralizing activity against Wu01 and Alpha, but only 7/9 showed neutralizing activity against Delta variant, while 5/9 showed neutralizing activity against Beta; neutralizing activity against the Omicron variant was abolished in 7/9 monoclonal antibodies. In conclusion, strong neutralizing activity against the Omicron variant can be induced by a booster dose of BNT162b2. However, follow-up studies will be required to determine the durability of the antibody neutralization activity against Omicron after the booster dose. Accordingly, Cameroni et al. [36] found that, on a panel of 44 neutralizing monoclonal antibodies, only six maintained potent neutralizing activity against Omicron. The monoclonal antibodies that retain neutralization recognize RBD antigenic sites that are conserved in Omicron. 

Table 1 shows the main studies on vaccine efficacy.

**Table 1:** Main studies and findings on vaccine efficacy.

Studies	Study Design	Vaccine Name	Vaccine Type	Main Inclusion/ Exclusion Criteria	No. of Subjects in Vaccine Group/No. of Subjects in Placebo Group	Outcome Type	Findings or Efficacy (%)
Saadat et al. [11]	Clinical research study	BNT162b2; mRNA-1273	mRNA vaccines	Healthcare workers previously enrolled in a hospital-wide serosurvey study and vaccinated with either the Pfizer/BioNTech or Moderna vaccine	*N* = 17 SARS-CoV-2 IgG antibody-negative (Ab-negative); *N* = 16 IgG-positive asymptomatic COVID-19 (asymptomatic); *N* = 26 IgG-positive with history of symptomatic COVID-19 (symptomatic)	Antibody responses	Healthcare workers with previous COVID-19 infection had higher antibody titer responses to a single dose of mRNA vaccine than those previously infected. Antibody titers started peaking at 7 days and achieved higher titers and neutralization in 14 days compared with Ab-negative volunteers.
Krammer et al. [12]	Short report	BNT162b2; mRNA-1273	mRNA vaccines	Subjects with and without documented pre- preexisting SARS-CoV-2, receiving their first vaccine dose in 2020	109 with/without documented preexisting SARS-CoV-2 (seronegative: 68, seropositive: 41)	Antibody responses	In seropositive subjects, the presence of post-vaccine antibody titers was comparable to or exceeded that in naïve subjects receiving two doses.
Parry et al. [13]	Clinical research study	BNT162b2 vaccine; ChAdOx1 nCoV-19 vaccine	mRNA vaccine and adenoviral vector vaccine	Subjects aged 80+ years who received a single dose of either BNT162b2 mRNA or ChAdOx1 adenovirus vaccine	165 participants *N* = 76 BNT162b2 mRNA *N* = 89 ChAdOx1 adenovirus vaccine	Immunogenicity (adaptive immune responses after 5 weeks)	Antibody responses against spike protein were detectable in 93% and 87% of mRNA or ChAdOx1 recipients, respectively, with median antibody titers of 19.3 and 19.6 U/mL (*p* = 0.41).
Sadoff et al. [14]	Randomized, double-blind, placebo-controlled, phase 3 trial	Ad26.COV2.S	Recombinant, adenoviral vector	SARS-CoV-2-negative adult subjects/RT-PCR-positive between days 1 and 14 or between days 1 and 28 were excluded from the analysis of cases with an onset at least 14 days after administration and at least 28 days after administration	per-protocol population: 39,321 SARS-CoV-2–negative participants *N* = 19,630 Ad26.COV2.S *N* = 19,691 placebo	Efficacy (against moderate to severe–critical coronavirus disease 2019 (COVID-19) with an onset at least 14 days and at least 28 days after administration)	Efficacy 66.9%.
Hall et al. [15]	Prospective cohort study	ChAdOx1 nCoV-19 (AZD1222) BNT162b2 mRNA	adenoviral vector vaccine and mRNA vaccine	Staff (aged ≥ 18 years) working in UK hospitals, assigned into either the positive cohort (antibody-positive or history of infection) or the negative cohort at the beginning of the follow-up period	23,324 participants *N* = 8203 assigned to the positive cohort *N* = 15,121 assigned to the negative cohort	Effectiveness	A single dose of BNT162b2 vaccine showed vaccine effectiveness of 70% (95% CI 55–85) 21 days after first dose and 85% (74–96) 7 days after two doses in the study population.
Fabiani et al. [16]	Retrospective cohort study	BNT162b2	mRNA vaccine	Adult healthcare workers (HCW) not infected/HCW infected with SARS-CoV-2 before the vaccination campaign, and HCW working outside hospitals	*N* = 6423 participants *N* = 1090 unvaccinated *N* = 147 vaccinated one dose *N* = 5186 vaccinated two doses	Effectiveness of one and two dose administration	84% in preventing asymptomatic and symptomatic SARS-CoV-2 infection (14–21 days from the first dose); 95% (7 days from the second dose).
Emary et al. [20]	Single-blind, multicenter, randomized phase 2/3 trial assessing the safety and efficacy of the ChAdOx1 nCoV-19 vaccine	ChAdOx1 nCoV-19 (AZD1222)	Adenoviral vector vaccine	Only participants in efficacy cohorts (*n* = 8534) were included; adults (≥18 years), with potentially high SARS-CoV-2 exposure, such as those in health and social care settings, enrolled at 19 study sites	8534 subjects randomly (1:1) assigned to receive standard-dose ChAdOx1 nCoV-19 vaccine (5 × 10^10^ viral particles) or a meningococcal group A, C, W, and Y conjugate vaccine (MenACWY) as control; 2773 subjects received a low-dose vaccine (2.2 × 10^10^ viral particles) as their first dose or control	The primary outcome: post hoc analysis of the efficacy of the adenoviral vector vaccine, ChAdOx1 nCoV-19 (AZD1222), against symptomatic COVID-19 disease for B.1.1.7	70.4% (95% CI 43.6–84.5%) efficacy against symptomatic NAAT positive infection for B.1.1.7 and 81.5% (67.9–89.4%) for non-B.1.1.7 lineage.
Voysey et al. [26]	Three single-blind randomized controlled trials—one phase 1/2 study in the UK (COV001), one phase 2/3 study in the UK (COV002), and one phase 3 study in Brazil (COV003)—and one double-blind phase 1/2 study in South Africa (COV005)	ChAdOx1 nCoV-19	Adenoviral vector vaccine	Participants in three single-blind randomized controlled trials	24,422 participants were recruited and vaccinated across the four studies, of whom 17,178 were included in the primary analysis (8597 receiving ChAdOx1 nCoV-19 and 8581 receiving control vaccine)	Virologically confirmed symptomatic COVID-19 disease, defined as a nucleic acid amplification test (NAAT)-positive swab combined with at least one qualifying symptom	Efficacy after a single standard dose of vaccine from day 22 to day 90 after vaccination was 76.0% (59.3–85.9%). Protection did not wane during this initial 3 month period.
Kuodi et al. [27]	A cross-sectional study nested in a prospective longitudinal cohort study	Mainly the BNT162b2	mRNA vaccine	All subjects (age ≥ 18 year) tested for SARS-CoV-2 infection by RT-PCR between 15 March 2020 and 15 November 2021 in the three major government hospitals in northern Israel were eligible	951 infected and 2437 uninfected subjects	Health outcomes according to vaccination and infection status	Fatigue (22%), headache (20%), weakness (13%), and persistent muscle pain (10%) were the most common symptoms. Subjects receiving two doses are less likely than unvaccinated individuals to report any of these symptoms by 64%, 54%, 57%, and 68%, respectively (Risk ratios: 0.36, 0.46, 0.43, 0.32, *p* < 0.04 in the listed sequence).
Choi et al. [29]	(1) mRNA-1273 booster open-label phase: booster dose of 50 µg mRNA-1273 (2) mRNA-1273 variant booster open-label phase: booster dose of mRNA-1273.351 20 or 50 µg or mRNA-1273.211 50 µg	mRNA-1273 mRNA-1273.351 mRNA-1273.211	mRNA vaccines	(1) Inclusion in the mRNA-1273 booster phase: only participants receiving 100 µg doses of mRNA-1273 in the P201 study were included in this analysis (2) Inclusion in the mRNA-1273 variant booster phase: participants must have been enrolled in the mRNA-1273-P301 COVE (NCT04470427) study and received two doses of mRNA-1273, with their second dose ≥6 months before enrollment in P201	(1) *N* = 600 randomized 1:1:1 to placebo or 50 or 100 µg mRNA-1273 in phase 2 study; *n* = 587 received 2 injections in the blinded phase; *n* = 558 proceeded to mRNA-1273 booster phase and offered option of unblinding at participant decision visit (2) *N* = ~30,400 randomized 1:1 to placebo or 100 µg mRNA-1273 in phase 3 COVE study; *n* = ~29,300 completed blinded phase; *n* = ~28,600 proceeded to open-label phase and offered option of unblinding at participant decision visit	Exploratory interim analysis of the preliminary safety and immunogenicity of single booster doses of mRNA-1273 (50 µg), modified mRNA-1273.351 and multivalent mRNA-1273.211	Boosters increased neutralization titers against VOCs and VOIs (B.1.351, P.1., and B.1.617.2), equivalent to peak titers measured after the primary vaccine series against wildtype D614G virus, and superior against some VOIs.
Patalon et al. [31]	Case–control study	BNT162b2	mRNA vaccine	306,710 members of Maccabi Healthcare Services (age ≥40 years; 55% female), receiving either 2 or 3 doses of BNT162b2 vaccine and without a positive PCR test result for SARS-CoV-2 before the follow-up period	500,232 PCR tests performed, 227,380 in subjects receiving 2 doses and 272,852 in those receiving 3 doses, with 14.989 (6.6%) and 4941 (1.8%) positive test results in each group, respectively	Retrospective study evaluating additional protection of a third booster dose of the BNT162b2 vaccine compared with a 2-dose regimen over the short term	Estimated odds ratio of 0.14 (95% CI, 0.13–0.15) 28 to 65 days following receipt of the booster (86% reduction in the odds of testing positive for SARS-CoV-2).
Doria-Rose et al. [34]	Laboratory study	mRNA-1273	mRNA vaccines	Participants receiving two doses of mRNA-1273	Serum samples from 30 vaccine recipients 4 weeks after second dose of mRNA-1273 in the phase 3 COVE study (NCT04470427) were assayed against D614G, Beta, or Omicron spike of SARS-CoV-2 in two independent laboratories; in a separate analysis, sequential serum samples from the 7 participants vaccinated and boosted under EUA were tested against D614G, Beta, and Omicron	To assess potential risk of Omicron variant to existing vaccines, serum samples from mRNA-1273 vaccine recipients were tested for neutralizing activity against Omicron and compared to neutralization titers against D614G and Beta	Neutralizing titers to Omicron were 49–84 times lower than neutralization titers to D614G after 2 doses of mRNA-1273, leading to an increased risk of symptomatic breakthrough infections. A booster dose of mRNA-1273 increases Omicron neutralization titers and may substantially reduce this risk.

## 5. Adverse Events

Greinacher et al. [37] described the cases of thrombocytopenia after the ChAdOx1 nCov-19 vaccination. Specifically, the clinical and laboratory characteristics of 11 patients in Germany and Austria who developed thrombosis or thrombocytopenia after vaccination were evaluated.

The first case was a 49 year old health worker, previously in good health, who had received the first dose of ChAdOx1 nCov-19 (AstraZeneca) in mid-February 2021. In the following days, she reported minor symptoms (fatigue, myalgia, and headache), while from the fifth day, her symptoms were chills, fever, nausea, and epigastric disorders. Ten days later, she was admitted to a local hospital. Computed axial tomography revealed thrombosis of the splanchnic vein. Despite transfusions of red blood cells, platelets, and coagulation factors, the patient died on day 11, and the autopsy also revealed a cerebral venous thrombosis. As of 15 March 2021, in addition to the first case, 10 other patients, for which clinical data were available, had thrombotic complications that started from day 5 to day 16 after vaccination with ChAdOx1 nCov-19. Thrombotic events included nine patients with venous thrombosis, three patients with splanchnic vein thrombosis, three patients with pulmonary embolism, and four patients with other types of thrombosis; five out of 10 patients had more than one thrombotic event. Signs of disseminated intravascular coagulation were also found in five patients. Hypofibrinogenemia was found in four out of six patients with available fibrinogen levels. Venous or arterial thrombosis was described about 5–20 days after vaccination and may represent an adverse effect of vaccination when accompanied by thrombocytopenia. Evaluation of the presence of anti-heparin antibodies (PF4), which are normally found in people who develop thrombocytopenia as a reaction to heparin, could help confirm the diagnosis of thrombotic thrombocytopenia induced by the vaccine in subjects not receiving heparin therapy.

Recently, after more than 6.8 million doses have been administered in the United States of the Ad26.COV2.S vaccine (Janssen, Johnson & Johnson), the FDA [38] is reviewing data regarding six reported cases of a rare and severe type of thrombosis in women between the ages of 18 and 48 between days 6 and 13 after vaccination. At this time, these adverse events appear to be extremely rare.

The etiopathogenetic mechanism of thrombosis hypothesized by Kadkhoda [39] based on some experimental studies could be the formation of immune glycoproteins complexes constituted from antibodies (IgG) against the SARS-CoV-2 spike protein. These immune complexes could activate platelets through a receptor present on their surface, inducing their activation and their adhesion to endothelial cells, which in turn would trigger the production of a coagulation factor (Willebrand factor). Platelet adhesion to endothelial cells could itself be one of the causes of severe thrombocytopenia (amount of circulating platelets less than 150,000/mm^3^) observed in these cases.

Kadkhoda [39] speculates that if the vaccine is accidentally injected into blood vessels, adenoviruses are able to infect cells such as epithelial, endothelial, and fibroblast cells, which can release copious amounts of soluble spike glycoproteins, resulting in a relatively high level of “antigenemia” spike SARS-CoV-2. Considering these mechanisms, Kadkhoda suggests a possible reduction in the vaccine dose and avoiding the administration of such vaccines in patients with coagulopathies or thrombocytopenia.

Kowarz et al. [40] described a mechanism that could underlie thromboembolic events triggered by DNA-based vaccines but not by RNA vaccines. According to the authors, DNA-encoded mRNA could more easily encode spike protein variants with unknown fate and function. The soluble spike variant secreted could then bind to ACE2 at the endothelial cell surface, being recognized by the anti-spikes generated during immunization. Inflammatory reactions could occur locally through several mechanisms including antibody-dependent cell-mediated cytotoxicity (ADCC) and complement-dependent cytotoxicity (CDC).

## 6. Conclusions

Surely, previous SARS-CoV-2 infection induces effective immunity to future infections in most individuals, with levels of protection from symptomatic infection similar to those of newly licensed vaccines for working-age adults. Such immunity is also protective against reinfection from some variants. Further studies are underway on the longevity of antibody responses, as well as the efficacy of third vaccines against COVID-19 on reinfection rates as part of the challenge against new variants. 

To achieve maximum public health benefit against the Omicron variant, many countries decided to give the third dose to as many individuals as possible.

Despite the limitations of the studies conducted so far, results seem to confirm the efficacy of third dose administration, resulting in high efficacy and protection against COVID-19 symptoms, but new variants may require the administration of a fourth dose.

The spike of SARS-CoV-2 evolves faster than other genomic regions via both point mutations [41,42] and recombination [43]. Therefore, several evolutionary scenarios for the future trajectory of SARS-CoV-2 [44] show that this pandemic may not yet be over. Serious efforts should probably be made to develop new vaccines that also target other more stable genomic regions. Currently, some vaccines under development target the capsid nucleus, which is evolutionarily more stable [45]. The emergence of the Omicron variant with strong mutations in its spike highlights this need to develop a new generation of vaccines that target more stable regions, rather than having to vaccinate every few months for a new variant.

## Data Availability

Links to publicly analyzed archived datasets are reported in the references.

## References

[1] Ghasemiyeh P., Mohammadi-Samani S., Firouzabadi N., Dehshahri A., Vazin A. (2021). A focused review on technologies, mechanisms, safety, and efficacy of available COVID-19 vaccines. Int. Immunopharmacol..

[2] Lundstrom K. (2020). The Current Status of COVID-19 Vaccines. Front. Genome Ed..

[3] World Health Organization Draft Landscape and Tracker of COVID-19 Candidate Vaccines. https://www.who.int/publications/m/item/draft-landscape-of-covid-19-candidate-vaccines.

[4] Higdon M.M., Wahl B., Jones C.B., Rosen J.G., Truelove S.A., Baidya A., Nande A.A., ShamaeiZadeh P.A., Walter K.K., Feikin D.R. (2021). A systematic review of COVID-19 vaccine efficacy and effectiveness against SARS-CoV-2 infection and disease. medRxiv.

[5] European Centre for Disease Prevention and Control (2021). Technical Report. Rollout of COVID-19 Vaccines in the EU/EEA: Challenges and Good Practice. Stockholm: ECDC. https://www.ecdc.europa.eu/sites/default/files/documents/Rollout-of-COVID-19-vaccinations-in-the-EU-EEA-challenges-good-practice-errata.pdf.

[6] Bar-On Y.M., Goldberg Y., Mandel M., Bodenheimer O., Freedman L., Kalkstein N., Mizrahi B., Alroy-Preis S., Ash N., Milo R. (2021). Protection of BNT162b2 Vaccine Booster against Covid-19 in Israel. N. Engl. J. Med..

[7] GOV.UK JCVI Interim Advice: Potential COVID-19 Booster Vaccine Programme Winter 2021 to 2022. https://www.gov.uk/government/publications/jcvi-interim-advice-on-a-potential-coronavirus-covid-19-booster-vaccine-programme-for-winter-2021-to-2022/jcvi-interim-advice-potential-covid-19-booster-vaccine-programme-winter-2021-to-2022.

[8] European Medicines Agency International Regulators’ Recommendations on COVID-19 Vaccines and the Omicron Variant. https://www.ema.europa.eu/en/news/international-regulators-recommendations-covid-19-vaccines-omicron-variant.

[9] International Coalition of Medicines Regulatory Authorities (ICMRA) ICMRA COVID-19 Omicron Variant Workshop. https://www.icmra.info/drupal/en/covid-19/12january2022.

[10] European Centre for Disease Prevention and Control COVID-19 Vaccine Tracker. https://qap.ecdc.europa.eu/public/extensions/COVID-19/vaccine-tracker.html#uptake-tab12january2022.

[11] Saadat S., Tehrani Z.R., Logue J., Newman M., Frieman M.B., Harris A.D., Sajadi M.M. (2021). Binding and Neutralization Antibody Titers After a Single Vaccine Dose in Health Care Workers Previously Infected With SARS-CoV-2. JAMA.

[12] Krammer F., Srivastava K., Simonet V., the PARIS team (2021). Robust spike antibody responses and increased reactogenicity in seropositive individuals after a 2 single dose of SARS-CoV-2 mRNA vaccine. medRxiv.

[13] Parry H.M., Bruton R., Tut G., Ali M., Stephens C., Faustini S., Huissoon A., Meade R., Brown K., Amirthalingam G. (2021). Single Vaccination with BNT162b2 or ChAdOx1 in Older People Induces Equivalent Antibody Generation but Enhanced Cellular Responses after ChAdOx1. SSRN.

[14] Sadoff J., Gray G., Vandebosch A., Cárdenas V., Shukarev G., Grinsztejn B., Goepfert P.A., Truyers C., Fennema H., Spiessens B. (2021). Safety and Efficacy of Single-Dose Ad26.COV2.S Vaccine against Covid-19. N. Engl. J. Med..

[15] Hall V.J., Foulkes S., Saei A., Andrews N., Oguti B., Charlett A., Wellington E., Stowe J., Gillson N., Atti A. (2021). COVID-19 vaccine coverage in health-care workers in England and effectiveness of BNT162b2 mRNA vaccine against infection (SIREN): A prospective, multicentre, cohort study. Lancet.

[16] Fabiani M., Ramigni M., Gobbetto V., Mateo-Urdiales A., Pezzotti P., Piovesan C. (2021). Effectiveness of the Comirnaty (BNT162b2, BioNTech/Pfizer) vaccine in preventing SARS-CoV-2 infection among healthcare workers, Treviso province, Veneto region, Italy, 27 December 2020 to 24 March 2021. Eurosurveillance.

[17] World Health Organization Tracking SARS-CoV-2 Variants. https://www.who.int/en/activities/tracking-SARS-CoV-2-variants/.

[18] Davies N.G., Abbott S., Barnard R.C., Jarvis C.I., Kucharski A.J., Munday J.D., Pearson C.A.B., Russell T.W., Tully D.C., Washburne A.D. (2021). Estimated transmissibility and impact of SARS-CoV-2 lineage B.1.1.7 in England. Science.

[19] Kemp S.A., Collier D.A., Datir R.P., Ferreira I.A.T.M., Gayed S., Jahun A., Hosmillo M., Rees-Spear C., Mlcochova P., Lumb I.U. (2021). SARS-CoV-2 evolution during treatment of chronic infection. Nature.

[20] Emary K.R.W., Golubchik T., Aley P.K., Ariani C.V., Angus B., Bibi S., Blane B., Bonsall D., Cicconi P., Charlton S. (2021). Efficacy of ChAdOx1 nCoV-19 (AZD1222) vaccine against SARS-CoV-2 variant of concern 202012/01 (B.1.1.7): An exploratory analysis of a randomised controlled trial. Lancet.

[21] Subbarao S., Warrener L.A., Hoschler K., Perry K.R., Shute J., Whitaker H., O’Brien M., Baawuah F., Moss P., Parry H. (2021). Robust antibody responses in 70–80-year-olds 3 weeks after the first or second doses of Pfizer/BioNTech COVID-19 vaccine, United Kingdom, January to February 2021. Eurosurveillance.

[22] PHE Monitoring of the Effectiveness of COVID-19 Vaccination. https://www.gov.uk/government/publications/phe-monitoring-of-the-effectiveness-of-covid-19-vaccination.

[23] Dagan N., Barda N., Kepten E., Miron O., Perchik S., Katz M.A., Hernán M.A., Lipsitch M., Reis B., Balicer R.D. (2021). BNT162b2 mRNA Covid-19 Vaccine in a Nationwide Mass Vaccination Setting. N. Engl. J. Med..

[24] Edara V.V., Hudson W.H., Xie X., Ahmed R., Suthar M.S. (2021). Neutralizing Antibodies Against SARS-CoV-2 Variants After Infection and Vaccination. JAMA.

[25] Hung I.F.N., Poland G.A. (2021). Single-dose Oxford–AstraZeneca COVID-19 vaccine followed by a 12-week booster. Lancet.

[26] Voysey M., Costa Clemens S.A., Madhi S.A., Weckx L.Y., Folegatti P.M., Aley P.K., Angus B., Baillie V.L., Barnabas S.L., Bhorat Q.E. (2021). Single-dose administration and the influence of the timing of the booster dose on immunogenicity and efficacy of ChAdOx1 nCoV-19 (AZD1222) vaccine: A pooled analysis of four randomised trials. Lancet.

[27] Kuodi P., Gorelik Y., Zayyad H., Wertheim O., Wiegler K.B., Jabal K.A., Dror A.A., Nazzal S., Glikman D., Edelstein M. (2022). Association between vaccination status and reported incidence of post-acute COVID-19 symptoms in Israel: A cross-sectional study of patients tested between March 2020 and November 2021. medRxiv.

[28] Juno J.A., Wheatley A.K. (2021). Boosting immunity to COVID-19 vaccines. Nat. Med..

[29] Choi A., Koch M., Wu K., Chu L., Ma L., Hill A., Nunna N., Huang W., Oestreicher J., Colpitts T. (2021). Safety and immunogenicity of SARS-CoV-2 variant mRNA vaccine boosters in healthy adults: An interim analysis. Nat. Med..

[30] Shroff R.T., Chalasani P., Wei R., Pennington D., Quirk G., Schoenle M.V., Peyton K.L., Uhrlaub J.L., Ripperger T.J., Jergović M. (2021). Immune responses to two and three doses of the BNT162b2 mRNA vaccine in adults with solid tumors. Nat. Med..

[31] Patalon T., Gazit S., Pitzer V.E., Prunas O., Warren J.L., Weinberger D.M. (2022). Odds of Testing Positive for SARS-CoV-2 Following Receipt of 3 vs 2 Doses of the BNT162b2 mRNA Vaccine. JAMA Intern. Med..

[32] ClinicalTrials.gov A Study to Evaluate the Immunogenicity and Safety of mRNA-1273.211 Vaccine for COVID-19 Variants. https://clinicaltrials.gov/ct2/show/NCT04927065?cond=A+Study+to+Evaluate+the+Immunogenicity+and+Safety+of+mRNA-1273.211+Vaccine+for+COVID-19+Variants&draw=2&rank=1.

[33] Moderna New Details. Moderna Announces Preliminary Booster Data and Updates Strategy to Address Omicron Variant. https://investors.modernatx.com/news/news-details/2021/Moderna-Announces-Preliminary-Booster-Data-and-Updates-Strategy-to-Address-Omicron-Variant/default.aspx.

[34] Doria-Rose N., Shen X., Schmidt S., O’Dell S., McDanal C., Feng W., Tong J., Eaton A., Maglinao M., Tang H. (2021). Booster of mRNA-1273 Vaccine Reduces SARS-CoV-2 Omicron Escapefrom Neutralizing Antibodies. medRxiv.

[35] Gruell H., Vanshylla K., Tober-Lau P., Hillus D., Schommers P., Lehmann C., Kurth F., Sander L.E., Klein F. (2022). mRNA booster immunization elicits potent neutralizing serum activity against the SARS-CoV-2 Omicron variant. Nat. Med..

[36] Cameroni E., Bowen J.E., Rosen L.E., Saliba C., Zepeda S.K., Culap K., Pinto D., VanBlargan L.A., De Marco A., di Iulio J. (2021). Broadly neutralizing antibodies overcome SARS-CoV-2 Omicron antigenic shift. Nature.

[37] Greinacher A., Thiele T., Warkentin T.E., Weisser K., Kyrle P.A., Eichinger S. (2021). Thrombotic Thrombocytopenia after ChAdOx1 nCov-19 Vaccination. N. Engl. J. Med..

[38] FDA https://www.fda.gov/news-events/press-announcements/joint-cdc-and-fda-statement-johnson-johnson-covid-19-vaccine.

[39] Kadkhoda K. (2021). Post-adenoviral-based COVID-19 vaccines thrombosis: A proposed mechanism. J. Thromb. Haemost..

[40] Kowarz E., Krutzke L., Külp M., Streb P., Larghero P., Reis J., Bracharz S., Engler T., Kochanek S., Marschalek R. (2022). Vaccine-induced COVID-19 mimicry syndrome. eLife.

[41] Lalioti V., González-Sanz S., Lois-Bermejo I., González-Jiménez P., Viedma-Poyatos Á., Merino A., Pajares M.A., Pérez-Sala D. (2021). Immunolocalization studies of vimentin and ACE2 on the surface of cells exposed to SARS-CoV-2 Spike proteins. bioRxiv.

[42] Amicone M., Borges V., Alves M.J., Isidro J., Zé-Zé L., Duarte S., Vieira L., Guiomar R., Gomes J.P., Gordo I. (2021). Mutation rate of SARS-CoV-2 and emergence of mutators during experimental evolution. bioRxiv.

[43] Nikolaidis M., Markoulatos P., Van de Peer Y., Oliver S.G., Amoutzias G.D. (2022). The Neighborhood of the Spike Gene Is a Hotspot for Modular Intertypic Homologous and Nonhomologous Recombination in Coronavirus Genomes. Mol. Biol. Evol..

[44] Amoutzias G.D., Nikolaidis M., Tryfonopoulou E., Chlichlia K., Markoulatos P., Oliver S.G. (2022). The Remarkable Evolutionary Plasticity of Coronaviruses by Mutation and Recombination: Insights for the COVID-19 Pandemic and the Future Evolutionary Paths of SARS-CoV-2. Viruses.

[45] Dutta N.K., Mazumdar K., Gordy J.T. (2020). The Nucleocapsid Protein of SARS–CoV-2: A Target for Vaccine Development. J. Virol..

